# Canadian women in otolaryngology: head and neck surgery part 2—challenges in family planning, fertility, and lactation

**DOI:** 10.1186/s40463-023-00630-z

**Published:** 2023-05-23

**Authors:** Kendra Naismith, Khrystyna Ioanidis, Agnieszka Dzioba, S. Danielle MacNeil, Josee Paradis, Smriti Nayan, Julie E. Strychowsky, M. Elise Graham

**Affiliations:** 1 Schulich School of Medicine and Dentistry, Western University, London, Canada; 2 Department of Otolaryngology – Head and Neck Surgery, Western University and London Health Sciences Centre, London, ON Canada; 3 Division of Otolaryngology - Head and Neck Surgery, Cambridge Memorial Hospital, McMaster University, Hamilton, ON Canada

**Keywords:** Canadian otolaryngologists, Family planning, Lactation, Fertility, Gender

## Abstract

**Background:**

Previous literature demonstrates that female surgeons face difficulties in family planning, meeting breastfeeding goals, leadership and advancement opportunities. These issues have received limited attention in Canadian surgeons despite different maternity leave patterns compared to the general Canadian population. We sought to describe the experience of otolaryngologist-head and neck surgeons in family planning, fertility, and lactation and to identify the role of gender and career stage in these experiences.

**Methods:**

A RedCAP^®^ survey was disseminated to Canadian otolaryngology–head and neck surgeons and residents from March to May of 2021 through social media and the national listserv. This survey examined fertility, pregnancy losses, and infant feeding. Major independent variables include gender and career stage (faculty and resident). Dependent variables include respondent experiences with fertility, number of children, and length of parental leave. Responses were tabulated and presented descriptively to communicate the experience of Canadian otolaryngologists. Further, statistical comparisons such as chi-square and t-tests were employed to identify relationships between these variables. Thematic analysis was conducted for narrative comments.

**Results:**

We received 183 completed surveys (22% response rate). 54% of females versus 13% of males agreed that career influenced their ability to have children (*p* = 0.002). 74% of female respondents without children have concerns about future fertility compared to 4% of men (*p* < 0.001). Furthermore, 80% of women versus 20% of men have concerns about future family planning (*p* < 0.001). The average maternity leave was 11.5 weeks for residents, and 22.2 weeks for staff. Additionally, significantly more women than men stated that maternity leave impacted advancement opportunities (32% vs. 7%) and salary/remuneration (71% vs. 24%) (*p* < 0.001). Over 60% of those choosing to pump breastmilk at work reported having inadequate time, space, and breastmilk storage. In total, 62% of breastfed infants were receiving breastmilk at 1 year.

**Conclusion:**

Canadian female otolaryngologists-head and neck surgeons face challenges in family planning, ability to conceive, and breastfeeding. Focused effort is required to provide an inclusive environment that helps all otolaryngologists-head and neck surgeons achieve both their career and family goals, regardless of gender or career stage.

**Graphical Abstract:**

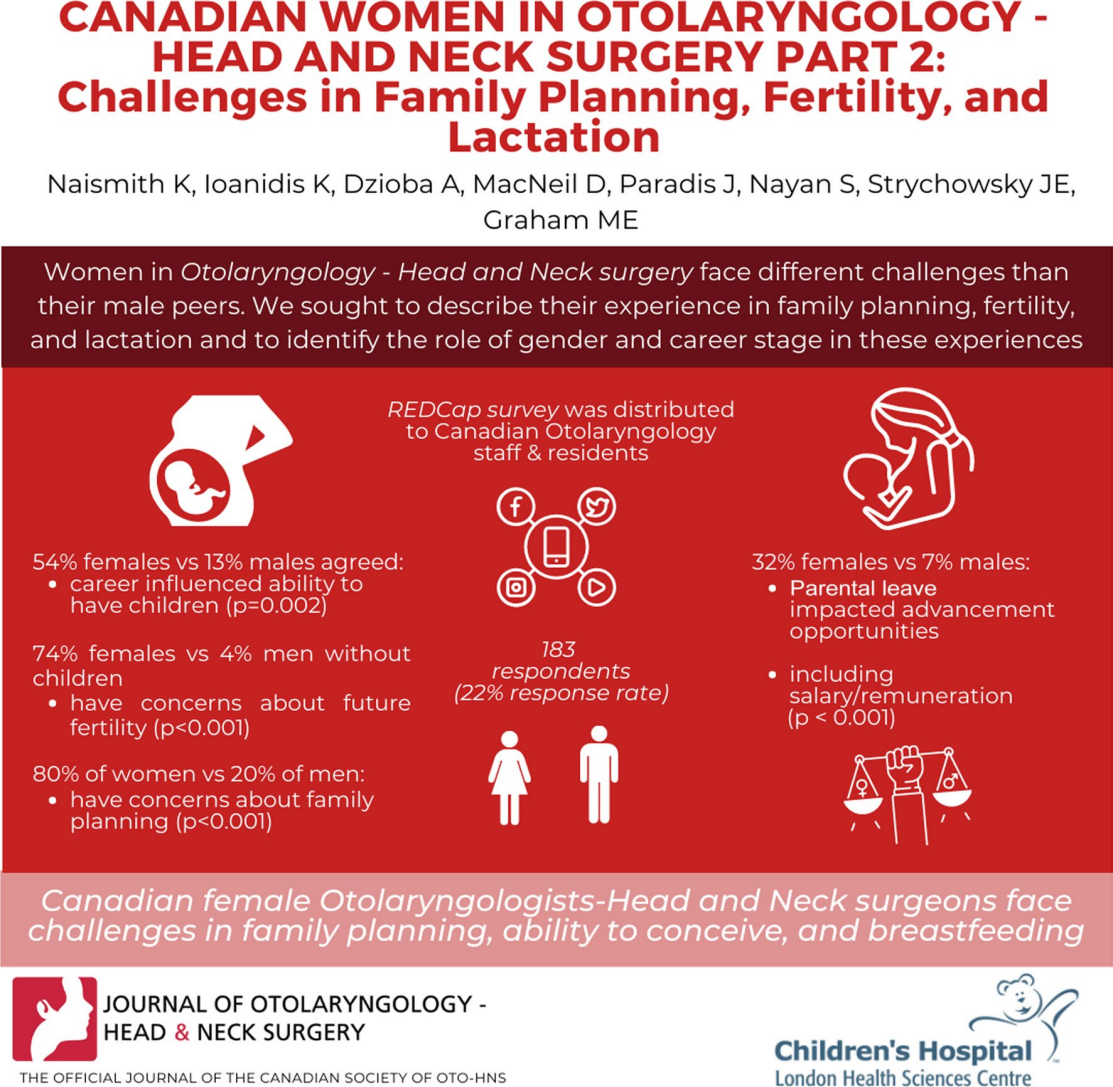

**Supplementary Information:**

The online version contains supplementary material available at 10.1186/s40463-023-00630-z.

## Background

Women in surgical disciplines face different challenges than their male peers. Previous research describing these challenges has largely originated in the United States. One study exploring this topic found that 32% of female surgeons experienced difficulty with fertility [1]. Within this study, otolaryngologists-head and neck surgeons were found to have the highest rate of infertility among the surgical specialties. [1]. Furthermore, female surgeons use fertility services and assistive reproductive technologies with higher frequency than the general population [1]. It is clear that a surgical career influences decisions around family planning and may impact fertility, though this has not been explored in all populations and surgical disciplines.

Fertility is not the only challenge faced by female surgeons. Data characterizing average parental leave in Canadian physicians is scarce. A recent study by Augustine et al. [2] investigated pregnancy and parental leave patterns in Canadian plastic surgery trainees and staff. This study found that significantly more residents and recent graduates (~ 65%) took a maternity leave of at least 6 months compared to approximately 15% of staff. In contrast, the 2010 Survey of Young Canadians found that Canadian women outside of Quebec take an average of 44 weeks of parental leave, while men take 2.4 weeks on average [3]. The American maternity leave norms are much less, where companies of 50 or more employees are legally required to provide only 12 weeks of unpaid leave [4].

A 2020 study of American women in otolaryngology-head and neck surgery by Lawlor et al. [5] found that 62% of women took more than 6 weeks of maternity leave, while 30% took less than 6 weeks. Of those women that took leave, 14% were on leave for less than 3 weeks [5]. Furthermore, 30% reported that maternity leave impacted their opportunities for career advancement and 40% stated that maternity leave affected their salary. The decision to pursue leadership positions was also impacted by having a child for 43% of respondents [5]. To date, there is very limited data outside of North America which would allow a more global assessment of the gender landscape of otolaryngology.

Because of the profound difference in pregnancy and parental leave norms between Canada and the United States, we postulate that the experience of Canadian otolaryngologists-head and neck surgeons may be different than their American counterparts. Shorter parental leave in surgeons as compared to the rest of the Canadian population may influence feeding choices, for example. The World Health Organization recommends exclusive breastfeeding for 6 months, with continued breastfeeding for 2 years or beyond [6]. Maternity leave timing may influence the ability to achieve this recommendation, and early return to work might increase difficulties faced by breastfeeding parents. For example, a survey of 170 female Canadian physicians investigating lactation supports found that 73% did not have adequate space, 68% did not have enough time, and 63% did not have access to adequate storage for pumped milk [7]. Thus far, the unique challenges faced by female otolaryngologists-head and neck surgeons in Canada has not been explored with regards to fertility, parental leave and lactation. In this two-part study, Canadian otolaryngologists were surveyed on the influence of gender on career progression in the workplace (Part I) and family, fertility, and lactation (Part II). The present paper (Part II in the manuscript series) reports on the influence of gender identity and career stage on experiences of fertility, parental leave and lactation.

## Methods

### Data collection

Institutional ethics review board approval for this study was obtained from Western University (REB# 118283). A survey was designed to address a variety of topics including demographics, fertility, pregnancy loss, parental leave and infant feeding (Additional file 1: Appendix 1). The survey was administered through REDCap (Version 11.1.13. Copyright © 2020 REDCap) to Canadian otolaryngologist–head and neck surgeons in active practice or retired, as well as trainees in accredited Canadian Otolaryngology-Head and Neck Surgery residency programs. Staff and trainees in specialties other than otolaryngology-head and neck surgery were excluded. There were approximately 838 eligible participants in Canada to whom the survey was distributed through the Canadian Society of Otolaryngology–Head and Neck Surgery (CSOHNS) national list-serv (551 consultants, 118 with emeritus status, and 169 residents). The survey was emailed at study opening, followed by a reminder email after a three-week time interval. The survey was made available in French and English from March to May 2021 and was further promoted through multiple social media platforms including Facebook^®^, Instagram^®^ and Twitter^®^. Survey responses were anonymous.

### Outcome measures

Survey questions are detailed in Additional file 1: Appendix 1. Categorial responses were collected for demographics, including gender identity, race/ethnicity, marital status, number of children, practice setting, subspecialty, etc. Questions regarding fertility, pregnancy and pregnancy complications, maternity leave, and lactation experience were evaluated using categorical responses (example, “have you accessed fertility services” (yes/no) and Likert scales assessing the level of agreement with statements (example, “Training/practice influenced my decision to have children” (disagree, somewhat disagree, neutral, somewhat agree, agree”), as appropriate. Optional free text responses were included to allow respondents to expand upon experiences. Open ended questions allowed qualitative analysis of the experiences of participants in the domains of family planning, fertility, pregnancy and lactation. Examples of these included “In your own words, please describe your experience pumping breastmilk at work”; and “please provide additional comments on how maternity/paternity leave has impacted advancement at work”.

### Data analysis

Responses with exclusively demographic data or without any demographic data were removed. Partially completed surveys (either partial demographics or partial responses to subsequent questions) were included in the analysis. Descriptive statistics including means and standard deviations were used to summarize demographic information such as age, number of children and age at birth of first child, and timeframes such as length of time spent on parental leave or breastfeeding. Frequencies were reported and evaluated for survey responses including Likert scales for degrees of agreement with statements regarding fertility, family planning, pregnancy and breastfeeding. Gender and career differences in study outcomes were explored with chi-squared tests to compare distributions amongst categorical responses and independent sample t-tests were employed for continuous variables. We implemented an alpha level of 0.05 to determine statistical significance. Data was processed and analyzed using R [8].

Open ended responses (qualitative data) were analyzed using thematic analysis. An inductive process was used for this with coding based on each question. This was performed by one researcher (KN) and themes consolidated with a second researcher (KI).

## Results

### Demographics

At survey closure, a total of 183 completed surveys were received, representing 22% of the Canadian society membership [838 members with 205 (24%) women]. Eighty-three (45%) respondents self-identified as female (40% response rate) and 100 (55%) as male (16% response rate). Residents made up 34% of respondents, and the remaining 66% were attending staff. There were no responses from retired otolaryngologists-head and neck surgeons. Respondents represented a variety of specialties, year of residency training, and years of practice experience. One hundred and one respondents (62%) had children, whereas 62 did not (38%) (Table 1). Among women without children, 11% have been pregnant in the past and 78% plan to have children in the future.

**Table 1:** Respondent demographics (n = 183)

Demographic	n (%)
*Gender*	
Female	83 (45.4)
Male	100 (54.6)
*Age*	
< 30	23 (13.2)
30–39	76 (43.7)
40–49	38 (21.8)
50–59	26 (14.8)
60 +	11 (6.3)
Ethnicity*	
Asian—East	24 (12.1)
Asian—South	14 (7.1)
Asian—Southeast	4 (2.0)
Black—African	1 (0.5)
First Nations	1 (0.5)
Latin American	1 (0.5)
Metis	1 (0.5)
Middle Eastern	13 (6.6)
White—European	32 (16.1)
White—North American	95 (48.0)
Mixed heritage	3 (1.5)
Other	4 (2.0)
Prefer not to answer	4 (2.0)
Do not know	1 (0.5)
*Province/territory*	
Alberta	21 (11.9)
British Columbia	18 (10.1)
Manitoba	14 (7.9)
New Brunswick	4 (2.2)
Newfoundland	1 (0.6)
Nova Scotia	21 (11.9)
Ontario	77 (43.5)
Prince Edward Island	1 (0.6)
Quebec	17 (9.6)
Saskatchewan	3 (1.7)
Northwest territories	0 (0)
Nunavut	0 (0)
Yukon	0 (0)
*Practice stage*	
Resident/fellow	62 (34.1)
Attending staff	120 (65.9)
Retired	0 (0)
*Resident PGY year*	
PGY 1	10 (16.1)
PGY 2	10 (16.1)
PGY 3	11 (17.7)
PGY 4	13 (21.0)
PGY 5	11 (17.7)
Fellow	7 (11.3)
*Practice setting*	
Academic	105 (58.3)
Community	37 (20.6)
Community-academic	38 (21.1)
*Relationship status*	
Married/common-law	133 (72.7)
Single	21 (11.5)
Cohabiting	10 (5.5)
Divorced/separated	8 (4.4)
Widowed	0 (0)
Prefer not to specify	1 (0.6)
Other	4 (2.2)
*Children*	
Yes	101 (61.6)
No	62 (37.8)
Prefer not to answer	1 (0.6)

*Multiple responses allowed for ethnicity

### Fertility and Family Planning

**Fig. 1:**
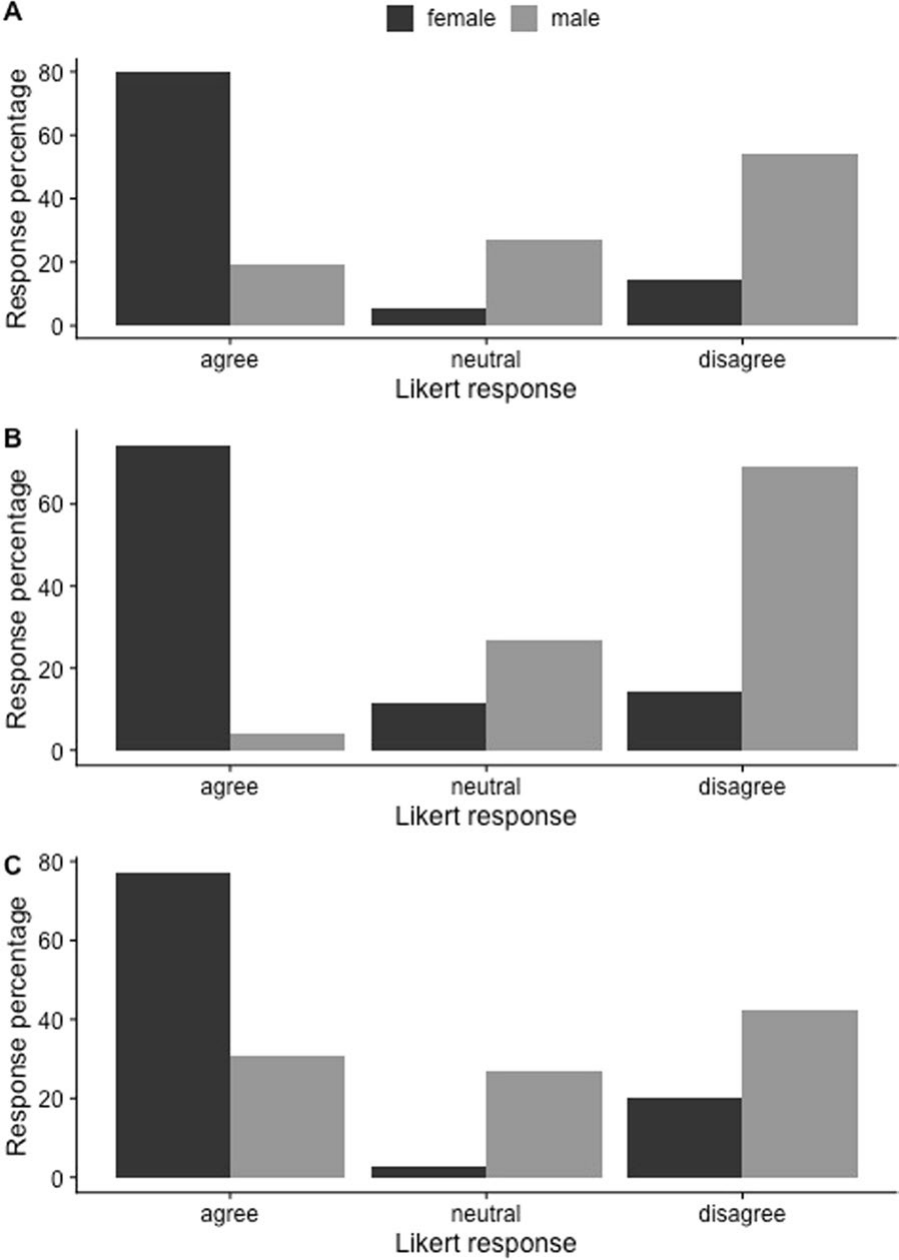
Female versus male response among respondents without children. **A** Likert response to the statement “I have concerns about future family planning” (*p* < 0.001). **B** Likert response to the statement “I have concerns about future fertility” (*p* < 0.001). **C** Likert response to the statement “I have concerns about future maternity/paternity leave” (*p* < 0.001). Likert responses “agree” and “somewhat agree” have been pooled to provide clarity as have the responses “somewhat disagree” and “disagree”

Among those without children, there was a gender difference concerning both the decision about *when* to have children and *ability* to have children. Three-quarters (75%) of female respondents believe that training/practice influenced their ability to have children, compared to 27% of men (*p* = 0.002). Similarly, there was a significant gender difference in concerns about future family planning, future fertility, and future parental leave. In all three of these categories, over 70% of female responses expressed some concern compared to 20%, 4% and 31% of men respectively (*p* < 0.001) (Fig. 1).

**Fig. 2:**
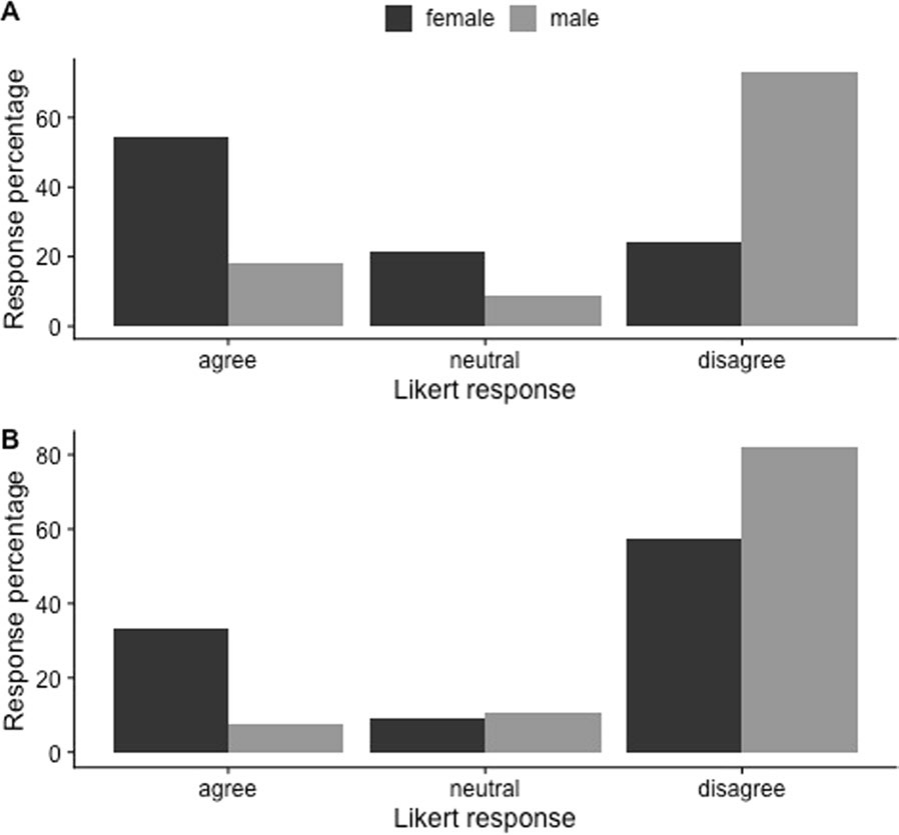
Female versus male response among respondents with children. **A** Likert response to the statement “Training/practice influenced the number of children I CHOSE to have” (*p* < 0.001). **B** Likert response to the statement “Training/practice influenced my ABILITY to conceive as many children as I wished to have” (*p* = 0.010). Likert responses “agree” and “somewhat agree” have been pooled to provide clarity as have the responses “somewhat disagree” and “disagree”

Among physicians with children, 54% of women versus 18% of men either “agreed” or “somewhat agreed” that their training/practice influenced the number of children they chose to have (*p* < 0.001). Similarly, a greater number of women with children (33%) versus men (8%) agreed or somewhat agreed that training/practice influenced their ability to conceive as many children as they wished to have (*p* = 0.01). (Fig. 2).

In thematic analysis of comments regarding fertility and family planning, nine of the 11 free text responses identified difficulty starting a family due to residency. This was endorsed by both male and female residents and attendings. Of the six female residents who provided a free text response, two expressed concerns about their fertility and family planning due to residency. One respondent expressed their concern by stating, "Training and early practice years definitely forces me to put my family planning "on-hold"… To me, being infertile as a direct result of a work/training-related reason would be the ultimate sacrifice that would weigh heavily on me."

### Pregnancy

Among physicians with children, the average number of children was 2.3. Interestingly, the median age of male and female respondents when their first child was born was the same at 32 years and there was a significant negative correlation between age of first child and number of children (r =  − 0.32; *p* = 0.002). With regards to achieving pregnancy, 20% of female respondents reported having accessed fertility services and 8% have accessed assistive reproductive technologies or in-vitro fertilization. Of the pregnancies reported by female respondents, 22% ended in miscarriage, 1.3% in stillbirth and 4.1% of pregnancies were terminated by therapeutic abortion (Table 2).

**Table 2:** Pregnancy outcomes in female respondents versus the Canadian population

Pregnancy outcome	Female respondents (%)	Canadian population (%)**	*P* value
Miscarriage	22	20	0.42
Stillbirth	1.3	0.85	0.70
Therapeutic abortion	4.1	17	< 0.001*

*Statistically significant

**Canadian 2020 population statistics of miscarriage [9], stillbirth [10] and therapeutic abortion [11]

When asked to provide a free text comment on experiences with miscarriage and pregnancy complications, 5 out of 10 described complications including difficulty conceiving, preterm delivery, pre-eclampsia, ectopic pregnancy and abortion. Three out of these 10 narrative comments also described distress associated with their experience. One respondent described their difficult experience by stating, "My miscarriage occurred while I was in clinic seeing patients. As I had not had any previous discussions with friends/family/colleagues/mentors regarding a miscarriage and being able to immediately process that this was a loss as well as the strong commitment to my family, I continued with the remainder of the clinic, didn't tell anyone until I got home (it was a Friday) and then grieved my loss with my partner and back to work Monday. In hindsight, I really wish someone had spoken more openly to me about these difficult times and that it is ok to take a step back from work briefly to actually be a ‘person’ and not just a ‘surgeon/doctor’ at all times. It was definitely hard."

### Parental leave and advancement

Among respondents with children, 97% of women and 46% of men had taken parental leave. During residency, female respondents went on maternity leave for an average of 11.5 weeks (SD = 6.1 weeks), versus male respondents who went on paternity leave for an average of 1.8 weeks (SD = 0.9 weeks) (*p* < 0.001). Among those in practice, the average length of parental leave was 22.2 weeks (SD = 11.3 weeks) for maternity leave compared to 2.9 weeks (SD = 1.7 weeks) for paternity leave (*p* < 0.001). 71% of female residents and 74% of female attendings indicated that they took as much leave as they intended. Factors with the greatest effect on maternity leave length included: support of partner/family/friends (moderate to strong effect for 61% of female respondents), concern about future opportunities (59%), financial concerns (54%) and difficulty finding coverage for practice (52%) (Table 3). Furthermore, there was a significant difference between male and female respondents with females more likely to cite a strong or moderate effect of the following factors on parental leave length: concern about future opportunities (*p* = 0.014), concern about stigma (*p* = 0.008), difficulty finding coverage for practice (*p* = 0.038), difficulty finding childcare (*p* = 0.004), and missing work/seeing patients (*p* = 0.027) (Table 3). There was only one significant difference in factors affecting length of parent leave between residents and attending staff, namely financial concerns; this was endorsed by 54% of attending staff and 0% of residents (*p* = 0.033).

**Table 3:** Factors influencing length of paternity leave versus maternity leave

Survey Response	Male	Female	*p* value
*Support of partner/family/friends*			0.600
Strong effect	19 (39%)	10 (36%)	
Moderate effect	12 (24%)	7 (25%)	
Neutral	6 (12%)	4 (14%)	
Minor effect	1 (2.0%)	3 (11%)	
No effect	11 (22%)	4 (14%)	
*Concern about losing skills*			0.130
Strong effect	3 (6.1%)	4 (14%)	
Moderate effect	7 (14%)	7 (24%)	
Neutral	11 (22%)	3 (10%)	
Minor effect	7 (14%)	8 (28%)	
No effect	21 (43%)	7 (24%)	
*Concern about future opportunities*			0.014*
Strong effect	4 (8.2%)	6 (21%)	
Moderate effect	7 (14%)	11 (38%)	
Neutral	6 (12%)	3 (10%)	
Minor effect	5 (10%)	3 (10%)	
No effect	27 (55%)	6 (21%)	
*Concern about stigma*			0.008*
Strong effect	2 (3.9%)	4 (14%)	
Moderate effect	12 (24%)	9 (31%)	
Neutral	8 (16%)	5 (17%)	
Minor effect	4 (7.8%)	7 (24%)	
No effect	25 (49%)	4 (14%)	
*Pressure from supervisors*			0.130
Strong effect	2 (3.9%)	1 (3.7%)	
Moderate effect	5 (9.8%)	5 (19%)	
Neutral	11 (22%)	5 (19%)	
Minor effect	3 (5.9%)	6 (22%)	
No effect	30 (59%)	10 (37%)	
*Pressure from colleagues*			*0.200*
Strong effect	1 (2.0%)	2 (6.9%)	
Moderate effect	6 (12%)	5 (17%)	
Neutral	4 (8.2%)	4 (14%)	
Minor effect	11 (22%)	10 (34%)	
No effect	27 (55%)	8 (28%)	
*Financial concerns*			0.400
Strong effect	11 (21%)	8 (29%)	
Moderate effect	15 (28%)	7 (25%)	
Neutral	4 (7.5%)	4 (14%)	
Minor effect	7 (13%)	5 (18%)	
No effect	16 (30%)	4 (14%)	
*Difficulty finding coverage for practice*			0.038*
Strong effect	5 (9.8%)	9 (33%)	
Moderate effect	13 (25%)	5 (19%)	
Neutral	9 (18%)	4 (15%)	
Minor effect	3 (5.9%)	4 (15%)	
No effect	21 (41%)	5 (19%)	
*Difficulty finding childcare*			0.004*
Strong effect	1 (2.0%)	4 (15%)	
Moderate effect	2 (4.0%)	5 (19%)	
Neutral	7 (14%)	6 (23%)	
Minor effect	8 (16%)	4 (15%)	
No effect	32 (64%)	7 (27%)	
*Miss working/seeing patients*			0.027*
Strong effect	3 (5.9%)	2 (6.9%)	
Moderate effect	9 (18%)	7 (24%)	
Neutral	7 (14%)	6 (21%)	
Minor effect	6 (12%)	9 (31%)	
No effect	26 (51%)	5 (17%)	
*Other*			0.016*
Strong effect	3 (30%)	0 (0%)	
Moderate effect	0 (0%)	2 (50%)	
Neutral	1 (10%)	2 (50%)	
Minor effect	0 (0%)	0 (0%)	
No effect	6 (60%)	0 (0%)	

*Statistically significant; Of the 14 “other” responses, 7 provided further explanation. Finishing residency on time was expressed by 4 respondents, two listed department norms and one described a family situation requiring return to work

**Fig. 3:**
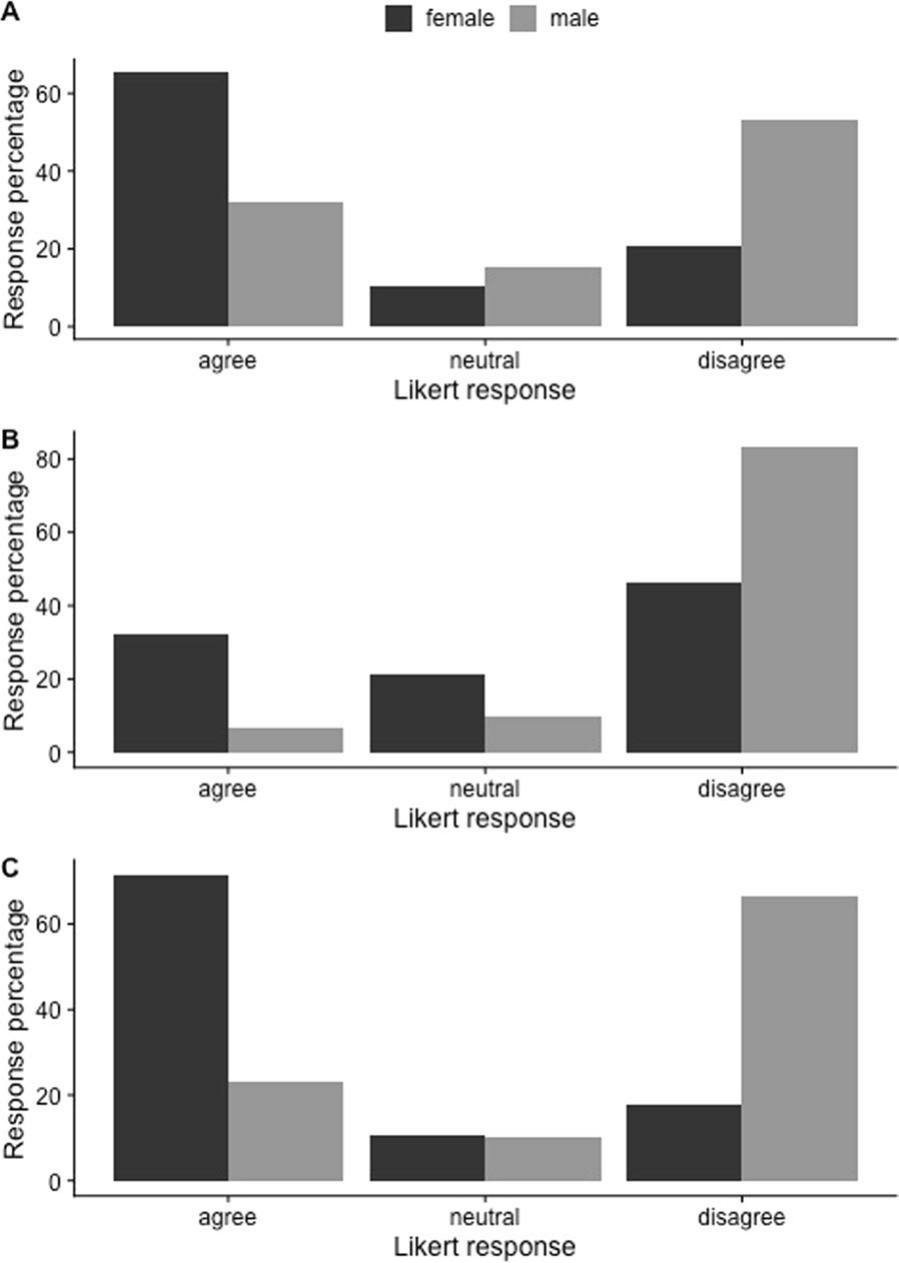
Female versus male responses regarding leadership and advancement. **A** Likert response to the statement “Having a child influenced my decision to pursue department or practice leadership roles” (*p* = 0.019). **B** Likert response to the statement “Maternity/paternity leave impacted my number of opportunities for career advancement” (*p* < 0.001). **C** Likert response to the statement “Maternity/paternity leave impacted my salary/remuneration” (*p* < 0.001). Likert responses “agree” and “somewhat agree” have been pooled to provide clarity as have the responses “somewhat disagree” and “disagree”

Pregnancy and parental leave were also found to influence advancement and leadership opportunities. Women were more likely than men to agree that having a child influenced their decision to pursue department or practice leadership roles (*p* = 0.019), that parental leave impacted the number of opportunities they had for career advancement (*p* < 0.001) and that parental leave impacted their salary/remuneration (*p* < 0.001) (Fig. 3).

In free text descriptions of how parental leave impacted advancement at work, four out of six female respondents cited time taken off as a concern, either resulting in having a limited amount of time to devote to reaching goals or requiring extra time to make up for their maternity leave. One respondent described their experience as "Difficulty in obtaining opportunities to gain skills after returning post leave- staff were surprised I did not lose skills and this occurred at each rotation—amounting to 3–4 weeks of advancement lost at each rotation until I could once again prove myself."

### Lactation

**Fig. 4:**
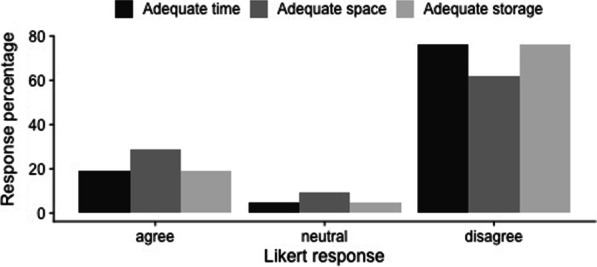
Likert response to pumping breastmilk at work. Likert responses of women who pumped breastmilk at work to the statements “I had adequate time to pump at work”, “I had adequate space to pump at work (clean, private, accessible)” and “I had adequate space for pumped breastmilk storage at work”. Likert responses “agree” and “somewhat agree” have been pooled to provide clarity as have the responses “somewhat disagree” and “disagree”

Of female respondents with children, 97% chose to breastfeed at least one child, with 43% choosing to pump breastmilk as a resident and 69% choosing to pump as an attending staff. By the end of one year, 62% of infants were receiving breastmilk either by direct feeds or pumped breastmilk. When asked to indicate on a Likert scale their experience with pumping breastmilk at work, 76% of women disagreed that they had adequate time to pump, 62% disagreed that they had adequate space for pumping and 76% disagreed that they had adequate space to store breastmilk at work (Fig. 4). Only 48% of respondents stated that they met their breastfeeding goals and 34% of respondents agreed that they felt supported in their decision to pump breastmilk at work. Two-thirds of women choosing to breastfeed experienced a breastfeeding complication (ex. blocked duct, mastitis) as a result of not being able to pump as frequently as needed.

In thematic analysis of the narrative responses to “In your own words, please describe your experience pumping breastmilk at work”, all 13 respondents (12 attendings and 1 resident) described an unconducive environment with 10 endorsing limited access to private rooms, 8 describing stress and discomfort pumping at work and 4 stating they had a lack of time. Additionally, 4 out of the 13 respondents described low milk production or early discontinuation of breastfeeding. One respondent described these challenges as follows, "I barely had enough time to pump and had to squeeze it in between other duties. Often eating my lunch while pumping and dictating in a public toilet. I recall stressing if a toilet was free. I never had time to pump more than 29–39 min a day and after a few weeks was mainly pumping just to maintain a bit of supply.".

## Discussion

Our large survey of Canadian otolaryngologists-head and neck surgeons explored many of the challenges related to family planning, fertility, and lactation. Three quarters of women in otolaryngology-head and neck surgery training and practice believe that their career impacted their fertility. Women were also disproportionately concerned about their future fertility, family planning and parental leave. Women in otolaryngology-head and neck surgery tended to take less parental leave than the average Canadian woman, with residents taking about 11.5 weeks and attending staff taking approximately 22.2 weeks. In contrast, Canadian woman on average take 44 weeks of maternity leave [3]. The length of parental leave of an otolaryngology-head and neck surgery staff or trainee, was impacted by a variety of factors, including concern about future opportunities, financial concerns and difficulty finding coverage. Because of this substantially shorter leave, otolaryngologists-head and neck surgeons face the additional challenge of finding childcare for an infant and increased need for lactation support in the workplace. In our survey, the majority of female respondents reported inadequate time, space, and storage for pumping breastmilk.

Canadian female otolaryngologists-head and neck surgeons are not alone in their concerns about fertility and family planning. Several American studies have noted the impact of a surgical career on fertility and pregnancy. Multiple studies have identified increased infertility amongst surgeons and an increased use of assistive reproductive technologies [1, 12, 13]. Surprisingly, otolaryngologists–head and surgeons were found to have the highest infertility rate among the surveyed surgical specialties [1]. The same study also found that American surgeons tend to have children later in life and fewer children overall compared to the American population [1]. Interestingly, another study focusing specifically at gynecological oncologists, noted that significantly more women than men reported that they would want to begin having children earlier if they were in a different career [12]. Finally, a 2021 study investigating fertility and pregnancy complications in multiple surgical specialties found that female surgeons experienced more pregnancy complications and losses than the general population [13]. In particular, female surgeons who operated more than twelve hours per week in their third trimester experienced a higher rate of major pregnancy complications compared to those who operated less than twelve hours per week [13]. Overall, these findings corroborate results of the present investigation. For many, being a woman in surgery means delaying childbirth and risking infertility or an unhealthy pregnancy.

In Canada, otolaryngologists-head and neck surgeons differ from the rest of the population with respect to fertility and family planning. There was a significant difference in maternal age with first child between our respondents, with an average maternal age of 32.7 years compared to the national Canadian average of 29.2 years in 2016 (*p* < 0.001) [14]. Many women in Canada receive employment insurance (EI) that offers 15 weeks of maternity leave and an additional 35 weeks of parental leave that can be shared between the parents [15]. This is a stark contrast to the average 11.5 week and 22.2 week maternity leaves taken by otolaryngology—head and neck surgery residents and staff in our survey, respectively. Most residents participate in EI and may receive additional financial support depending on their province [16]. Despite 50 weeks of maternity and parental leave coverage, residents tend to take far less time off. This is partly explained by one of the factors highlighted in several of the residents’ comments, namely “finishing residency on time.” The Royal College of Physicians and Surgeons of Canada provides a waiver of training for up to three months of leave within a five-year residency program such as Otolaryngology-Head and Neck Surgery [17]. A longer leave would require additional time and could impact graduation and possibly fellowship opportunities. Therefore, although EI provides financial support for approximately one year, the average resident maternity leave is just shy of 12 weeks. Some attendings may also benefit from EI depending on practice setting or if they decided to opt-in. For those staff who are not eligible for EI, each province provides some form of parental leave support, ranging from $1000 to $2000 per week for 17 to 26 weeks, depending on the province [18]. It is also important to note that, unlike residents, physicians often have ongoing expenses throughout their maternity leave, such as overhead and membership dues. This helps explain why 54% of attendings listed financial concerns as a factor impacting length of leave, whereas 0% of residents selected finances as a factor. Because otolaryngology-head and neck surgery residents and staff tend to take less maternity leave than the general Canadian population, they face unique struggles regarding childcare and breast feeding. This is likely even more pronounced compared to their American colleagues where a short maternity leave is commonplace.

Regarding lactation, the Canadian Charter protects the right to breastfeed under the right of gender equality [19] and the Canadian Human Rights Commission recommends employers accommodate a mother’s need to breastfeed or express milk in the workplace to avoid discrimination [20]. The provinces of British Columbia and Ontario further clarify the specific right to breastfeeding protection in the workplace [21, 22]. The Canadian Human Rights Commission “Pregnancy and Human Rights in the Workplace | A Guide for Employers” provides a plethora of suggestions to help employers accommodate breastfeeding women, including offering flexibility in working hours, allowing for longer or extra breaks and a private space for breastfeeding or pumping breastmilk [20]. The American Association of Family Physicians and the Association of Women Surgeons present these same recommendations and expand on them in a medical context. They recommend a private lactation facility that contains the necessary storage and possibly a computer station to provide an option for women to work while pumping [23, 24]. They also recommend providing a protected 30 min approximately every 3 h for feeding or expressing breastmilk [23, 24]. Along with provision of time and space, creating a culture that supports breastfeeding women is essential [23, 24].

We have identified a few limitations to our study. First, we recognize the presence of a selection bias. We captured 22% of the Canadian Society of Otolaryngology—Head and Neck Surgery membership, leaving close to 80% of the membership unrepresented in our results. Because of this, we cannot ensure that the opinions captured in our survey responses represent the entirety of the CSO membership. Similarly, all those who responded to the survey self-identified their gender as either male or female. We did not capture anyone whose experience of gender falls outside of the binary and are therefore unable to comment on their experience as an otolaryngologist–head and neck surgeon or trainee. Furthermore, our survey questions may not have provided adequate representation for everyone’s experience, for example those who have chosen adoption or who have blended families. Finally, we did not receive enough responses to adequately power the study to make comparisons across age groups. Because of this, we are unable to comment on any changes with regards to experiences of family planning, parental leave and lactation over time. Future directions include explorations of the differing experiences of otolaryngologists elsewhere in the world, as the existing literature including our own work originates in North America, and our findings may not be consistent with the experience of the global otolaryngology community.

## Conclusions

More work is required to provide women with an environment that is supportive of them and their family goals. Female otolaryngologists-head and neck surgeons tend to take less parental leave than the typical 44 weeks in Canada, requiring unique supports as they transition back to the workplace. We need to invite diverse voices into the discussion moving forward to provide effective and equitable solutions. Opening the conversation, normalizing having a family, parental leave and lactation along with providing protected breaks and private space for expressing breastmilk is essential in supporting otolaryngology-head and neck surgery staff and trainees, regardless of gender, in both their career and family goals.

## Supplementary Information

**Additional file 1: Appendix 1.** Survey.

## Data Availability

The complete survey is made available in Additional file 1: Appendix 1. Survey data can be made available upon request.

## Abbreviation

SD: Standard deviation
