# Supplementary material for: Canadian women in otolaryngology: head and neck surgery part 2—challenges in family planning, fertility, and lactation
Source: J Otolaryngol Head Neck Surg. 2023 May 23;52:39. doi: 10.1186/s40463-023-00630-z (PMC10207624; doi:10.1186/s40463-023-00630-z)
Supplement: Supplementary file 1 — Additional file 1: Appendix 1. Survey. [file 40463_2023_630_MOESM1_ESM.pdf]

Influence of gender identity on family planning and professional advancement in otolaryngology - head and neck surgery

Letter of Information and Consent

Project Title: Influence of gender identity on family planning and professional advancement in otolaryngology - head and neck surgery

Principal Investigator: Elise Graham, MD, FRCSC, Department of Otolaryngology-Head and Neck Surgery, Western University, London, ON

PARTICIPATION You are being invited to take part in a study surveying Canadian otolaryngologists-head and neck surgeons and otolaryngology-head and neck surgery residents about the influence of gender identity on family planning and professional advancement in otolaryngology-head and neck surgery. Mentorship, academic promotion, pregnancy, and maternity/paternity leave each have their unique challenges for female trainees and staff. However, there is a paucity of literature exploring these challenges and their effects on a Canadian surgeon's career. The present study seeks to obtain a better understanding of the connections between academic practice, family planning, fertility challenges and lactation concerns in Canadian otolaryngologists. In addition, a secondary objective of the survey is to identify gender specific patterns in practice choice and leadership positions among otolaryngologists-head and neck surgeons.

Completion of this survey will take approximately 10 minutes. Your participation in this survey is voluntary. You may refuse to take part in the research or exit the survey at any time without penalty. You are free to decline to answer any particular question you do not wish to answer for any reason. A reminder message with the survey link will be sent out to all eligible participants in three weeks-time. Your decision to take part in this study will not affect your employment in any way. Approximately 800 participants will be enrolled in this study.

BENEFITS You will receive no direct benefits from participating in this research study. However, your responses may help us learn more about connections gender, academic practice, family planning, and fertility challenges in Canadian otolaryngologists-head and neck surgeons. Results from this study may help highlight areas of the practice where support is lacking and identify work-life issues that need to be addressed.

RISKS With the exception of a potential risk of privacy breech, there are no foreseeable risks in participating in this study. CONFIDENTIALITY Your survey answers will be sent to a link at redcap.lawsonresearch.ca where data will be stored in a password protected electronic format. We will not be collecting identifying information such as your name, email address, or IP address. However, as part of this survey, we will be asking questions about your gender, years in practice/training, and sub-specialties. Your responses will remain anonymous. No one will be able to identify you or your answers, and no one will know whether or not you participated in the study. Your legal rights are not waived by agreeing to participate in this study. Study data will be retained for 15 years following study cessation, after which compiled data files will be destroyed and electronic study database dismantled. Western University Health Sciences Research Ethics Board and Lawson Quality Assurance and Education Program may require access to study records to monitor the conduct of the study and for quality assurance purposes. Results of the study will be published in a peer reviewed journal.

STUDY WITHDRAWAL

Survey questions can be skipped and the survey can be closed without submitting it. However, study withdrawal once the survey is submitted is not possible due to the anonymous nature of survey.

CONTACT If you have questions at any time about the study or the procedures, you may contact the research supervisor, Dr. Elise Graham via email at Elise.Graham@lhsc.on.ca.

If you feel you have not been treated according to the descriptions in this form, or that your rights as a participant in research have not been honored during the course of this project, or you have any questions, concerns, or complaints that you wish to address to someone other than the investigator, you may contact the Patient Experience Office at LHSC at 519 685-8500 ext. 52036 or access the online form at https://apps.lhsc.on.ca/?q=forms/patient-experience-contact-form.

ELECTRONIC CONSENT:

Clicking on the link to the survey below, indicates that

☐ You have read the above information

☐ You voluntarily agree to participate

☐ You are 18 years of age or older

Submission of the survey indicates your consent to participate.

What language would you like the survey in:

◯ English
◯ French

Influence of gender identity on family planning and professional advancement in otolaryngology - head and neck surgery

Please click "Next Page" to begin the survey.

Demographics

What is your age (in years)?

____

What is your gender identity?

- ◯ agender
- ◯ genderqueer or genderfliud
- ◯ man
- ◯ non-binary
- ◯ questioning or unsure
- ◯ two-spirit
- ◯ woman
- ◯ prefer not to disclose
- ◯ additional gender category ______

What is your sexual orientation?

- ◯ Aromantic
- ◯ Asexual
- ◯ Bisexual
- ◯ Fluid
- ◯ Gay
- ◯ Lesbian
- ◯ Pansexual
- ◯ Queer
- ◯ Questioning or unsure
- ◯ Same gender-loving
- ◯ Straight (heterosexual)
- ◯ Prefer not to disclose
- ◯ Additional category/identity no listed (please specify) ______

What pronouns do you use?

- ☐ he/him
- ☐ she/her
- ☐ they/their
- ☐ other, please specify ______

(select all that apply)

What is your race/ethnicity? (select all that apply)	☐ Asian - East (eg, Chinese, Japanese, Korean)<br>☐ Asian - South (eg, Indian, Pakistani, Sri Lankan)<br>☐ Asian - Southeast (eg, Malaysian, Filipino, Vietnamese)<br>☐ Black - African (eg, Ghanaian, Kenyan, Somali)<br>☐ Black - Caribbean (eg, Barbadian, Jamaican)<br>☐ Black - North American (eg, Canadian, American)<br>☐ First Nations<br>☐ Indian - Caribbean (eg, Guyanese with origins in India)<br>☐ Indigenous or Aboriginal not included elsewhere<br>☐ Inuit<br>☐ Latin American (eg, Argentinian, Chilean, Salvadoran)<br>☐ Metis<br>☐ Middle Eastern (eg, Egyptian, Iranian, Lebanese)<br>☐ White - European (eg, English, Italian, Portuguese, Russian)<br>☐ White - North American (eg, American, Canadian)<br>☐ Mixed heritage (eg, black African and white North American)<br>☐ Other please specify ______<br>☐ Prefer not to answer<br>☐ Do not know<br>(select all that apply)
What is your practice setting?	◯ Academic<br>◯ Community<br>◯ Community-Academic
What is your marital status?	◯ Married/Common-Law<br>◯ Single<br>◯ Cohabiting<br>◯ Divorced/Separated<br>◯ Widowed<br>◯ Prefer not to specify<br>◯ Other, please specify ______
What province are you located in?	◯ Alberta<br>◯ BC<br>◯ Manitoba<br>◯ New Brunswick<br>◯ Newfoundland<br>◯ Nova Scotia<br>◯ Ontario<br>◯ PEI<br>◯ Quebec<br>◯ Saskatchewan<br>◯ NWT<br>◯ Nunavut<br>◯ Yukon
What is your stage of practice?	◯ Resident/Fellow<br>◯ Attending staff<br>◯ Retired

What PGY year are you in?	◯ 1<br>◯ 2<br>◯ 3<br>◯ 4<br>◯ 5<br>◯ Fellow
Do you intend to subspecialize?	◯ No/General<br>◯ Facial Plastics<br>◯ Head and Neck Surgery<br>◯ Laryngology<br>◯ Otology<br>◯ Pediatric Otolaryngology<br>◯ Rhinology<br>◯ Undecided
How many years have you been in practice?	◯ Less than 5<br>◯ 5 - 9<br>◯ 10 - 14<br>◯ 15 - 19<br>◯ 20 or more
On average, how many days do you work (clinical and non-clinical) per week?	◯ 5 or more<br>◯ 3 - 4<br>◯ Less than 3
Did you complete additional fellowship/subspecialty training?	◯ Yes<br>◯ No
What is your primary subspecialty?	◯ General<br>◯ Facial Plastics<br>◯ Head and Neck Surgery<br>◯ Laryngology<br>◯ Otology<br>◯ Pediatric Otolaryngology<br>◯ Rhinology
What is your secondary specialty? (select all that appy)	☐ General<br>☐ Facial Plastics<br>☐ Head and Neck Surgery<br>☐ Laryngology<br>☐ Otology<br>☐ Pediatric Otolaryngology<br>☐ Rhinology<br>☐ None<br>(select all that apply)
What is your current academic rank?	◯ Lecturer<br>◯ Assistant Professor<br>◯ Associate Professor<br>◯ Full Professor<br>◯ Other, please specify ______<br>◯ None
How many years did it take to obtain this ranking? (enter number of years)	______________________________

Residency Training

What was the percentage of same gender co-residents over the duration of your residency?	◯ 0-20%<br>◯ 20-40%<br>◯ 40-60%<br>◯ 60-80%<br>◯ 80-100%
What was the percentage of same gender attending staff while in residency?	◯ 0-20%<br>◯ 20-40%<br>◯ 40-60%<br>◯ 60-80%<br>◯ 80-100%

Please state your agreement with the following statements about department/division leaders during residency:

	disagree	somewhat disagree	neutral	somewhat agree	agree	N/A
My department leaders were supportive of residents starting families	◯	◯	◯	◯	◯	◯
My department was supportive of time away for maternity leave	◯	◯	◯	◯	◯	◯
My department was supportive of time away for paternity leave	◯	◯	◯	◯	◯	◯
My department allowed me to take time away for family issues	◯	◯	◯	◯	◯	◯
My department had the same expectations of residents regardless of gender	◯	◯	◯	◯	◯	◯
Residents of all genders were evaluated fairly based on the same criteria	◯	◯	◯	◯	◯	◯
My program leaders treated all residents equally regardless of gender	◯	◯	◯	◯	◯	◯
The same leadership opportunities were open to everyone regardless of gender	◯	◯	◯	◯	◯	◯

Please state your agreement with the following statements about your co-residents during residency:

	disagree	somewhat disagree	neutral	somewhat agree	agree
My female co-residents were supportive of women starting families	◯	◯	◯	◯	◯
My male co-residents were supportive of women starting families	◯	◯	◯	◯	◯
My female co-residents were supportive of those taking time away for family issues	◯	◯	◯	◯	◯
My male co-residents were supportive of those taking time away for family issues	◯	◯	◯	◯	◯

How would you rate your residency experience overall?

◯ excellent
◯ very good
◯ good
◯ fair
◯ poor

What would you change about your residency program?

____

Leadership

What are your leadership roles (past and present)? (select all that apply)

☐ Department Chair/Chief
☐ Site Chief
☐ Division Chief
☐ Program Director
☐ Assistant Program Director
☐ Rotation Supervisor
☐ Other ______
☐ None
(select all that apply)

In your current department, what percentage of the following roles are held by women?

	0-20%	20-40%	40-60%	60-80%	80-100%
Physicians	◯	◯	◯	◯	◯
Department Chair/Chief	◯	◯	◯	◯	◯
Site Chief	◯	◯	◯	◯	◯
Division Chief	◯	◯	◯	◯	◯
Program Director	◯	◯	◯	◯	◯
Assistant Program Director	◯	◯	◯	◯	◯

Rotation Supervisor	○	○	○	○	○

Harrassment Harassment is a form of discrimination. It includes any unwanted physical or verbal behaviour that offends or humiliates you. Generally, harassment is a behaviour that persists over time. Serious one-time incidents can also sometimes be considered harassment.

Harassment occurs when someone:

makes unwelcome remarks or jokes about your race, religion, sex, age, disability or any other of the grounds of discrimination; threatens or intimidates you because of your race, religion, sex, age, disability or any other of the grounds of discrimination; makes unwelcome physical contact with you, such as touching, patting, or pinching. Canadian Human Rights Commission (https://www.chrc-ccdp.gc.ca/eng/content/what-harassment-1)

Did you experience harassment during residency?	○ Harassment free<br>○ Subtle undertones of harassment<br>○ Noticeable tones of harassment<br>○ Significant level of harassment<br>○ Unsure
Types of harassment experienced during residency (select all that apply)	☐ Verbal (non-sexual)<br>☐ Sexual harassment (verbal)<br>☐ Sexual harassment (physical)<br>☐ Racial/Ethnic harassment<br>☐ Physical harassment (non-sexual)<br>(select all that apply)
Who was responsible for the harassment? (select all that apply)	☐ Leaders in my department<br>☐ Colleagues/Other resident<br>☐ Patients or family members<br>☐ Ancillary Staff<br>☐ Administration<br>(select all that apply)
Did you experience harassment at work?	○ Harassment free<br>○ Subtle undertones of harassment<br>○ Noticeable tones of harassment<br>○ Significant level of harassment<br>○ Not sure
Types of harassment experienced post-residency/in practice (select all that apply)	☐ Verbal (non-sexual)<br>☐ Sexual harassment (verbal)<br>☐ Sexual harassment (physical)<br>☐ Racial/Ethnic harassment<br>☐ Physical harassment (non-sexual)<br>(select all that apply)
Who was responsible for the harassment? (select all that apply)	☐ Leaders in my department<br>☐ Colleagues<br>☐ Residents<br>☐ Patients or family members<br>☐ Ancillary Staff<br>☐ Administration<br>(select all that apply)
Please provide additional comments on your experience with harassment:	____

Work-Life Balance Please state your agreement with the following statements:

	disagree	somewhat disagree	neutral	somewhat agree	agree
I feel comfortable as a [gender_identity] at work	◯	◯	◯	◯	◯
It is easy managing work-life balance	◯	◯	◯	◯	◯
I am happy with my decision to be an otolaryngologist-head and neck surgeon	◯	◯	◯	◯	◯
I would recommend otolaryngology-head and neck surgery as a career to a young [gender_identity]	◯	◯	◯	◯	◯

Children, Lactation, and Parental Leave

Do you have children?	◯ yes<br>◯ no<br>◯ prefer not to answer
Have you ever been pregnant?	◯ Yes<br>◯ No<br>◯ Not applicable
Do you plan to have children in the future?	◯ yes<br>◯ no<br>◯ unsure
Did you/your partner experience any miscarriages?	◯ Yes, how many? ______<br>◯ No
Did you/your partner experience any stillbirths	◯ Yes, how many? ______<br>◯ No
Did you/your partner have any therapeutic abortions?	◯ Yes, how many? ______<br>◯ No
Did you/your partner have any abnormal prenatal screening during any pregnancy?	◯ Yes<br>◯ No
Please describe further your experience with miscarriage and pregnancy complication	____

Please state your level of agreement with the following statements:

	disagree	somewhat disagree	neutral	somewhat agree	agree
Training/practice influenced my decision to have children	◯	◯	◯	◯	◯
Training/practice influenced my decision about WHEN to have children	◯	◯	◯	◯	◯
Training/practice influenced my ABILITY to have children	◯	◯	◯	◯	◯
I have concerns about future family planning	◯	◯	◯	◯	◯
I have concerns about future fertility	◯	◯	◯	◯	◯
I have concerns about future maternity/paternity leave	◯	◯	◯	◯	◯

Have you accessed fertility services?
◯ Yes
◯ No

Have you used assistive reproductive technologies/IVF?
◯ Yes
◯ No

If you have experienced difficulty with conception, what was your underlying diagnosis (if given)?
____

Please provide additional comments regarding fertility and family planning:
____

How many children do you have?
____

How old were you when your first child was born?
____

When were your children born? (Check all that apply)
☐ Before medical school
☐ Medical school
☐ Residency/Fellowship
☐ Clinical Practice
☐ Other
(select all that apply)

Did you take maternity/paternity leave during medical school, residency or clinical practice?
◯ Yes
◯ No

On average, how much time did you take for maternity/paternity leave during medical school (please enter number of weeks)?
____

Did you take as much leave as you planned/intended during medical school? (y/n)
◯ Yes
◯ No

On average, how much time did you take for maternity/paternity leave during residency (please enter number of weeks)?
____

Did you take as much leave as you planned/intended during residency?	◯ Yes<br>◯ No
On average, how much time did you take for maternity/paternity leave during clinical practice (please enter number of weeks)?	__________________________________
Did you take as much leave as you planned/intended during clinical practice?	◯ Yes<br>◯ No
If you have a partner, did your partner take leave?	◯ yes<br>◯ no<br>◯ not applicable
On average, how long did your partner take leave? (please enter number of weeks)	__________________________________

Please state the extent to which the following factors influenced your length of leave:

	Did not affect	Minor affect	Neutral	Moderate affect	Strong affect	N/A
Support of partner/family/friends	◯	◯	◯	◯	◯	◯
Concern about losing skills	◯	◯	◯	◯	◯	◯
Concern about future opportunities	◯	◯	◯	◯	◯	◯
Concern about stigma	◯	◯	◯	◯	◯	◯
Pressure from supervisors	◯	◯	◯	◯	◯	◯
Pressure from colleagues	◯	◯	◯	◯	◯	◯
Financial concerns	◯	◯	◯	◯	◯	◯
Difficulty finding coverage for practice	◯	◯	◯	◯	◯	◯
Difficulty finding child care	◯	◯	◯	◯	◯	◯
Miss working/seeing patients	◯	◯	◯	◯	◯	◯
Other, please specify ______	◯	◯	◯	◯	◯	◯

Training/practice influenced the number of children I CHOSE to have?	◯ disagree<br>◯ somewhat disagree<br>◯ neutral<br>◯ somewhat agree<br>◯ agree
Training/practice influenced my ABILITY to conceive as many children as I wished to have?	◯ disagree<br>◯ somewhat disagree<br>◯ neutral<br>◯ somewhat agree<br>◯ agree
Did you access fertility services to help conceive a child?	◯ Yes<br>◯ No
Did you use assistive reproductive technologies?	◯ Yes<br>◯ No

Did you choose adoption?

◯ Yes
◯ No

If you had difficulty with conception, what was your underlying diagnosis?

____

Maternity/Paternity Leave and Advancement

On average, at how many weeks gestational age did you stop call?

____

Please indicate your agreement with the following statements:

	disagree	somewhat disagree	neutral	somewhat agree	agree	N/A
Colleagues were supportive of my pregnancy	◯	◯	◯	◯	◯	◯
Colleagues were supportive of my maternity/paternity leave	◯	◯	◯	◯	◯	◯
Having a child influenced my decision to pursue fellowship or additional post-residency education	◯	◯	◯	◯	◯	◯
Having a child influenced my decision to pursue department or practice leadership roles	◯	◯	◯	◯	◯	◯
Maternity/paternity leave impacted my number of opportunities for career advancement	◯	◯	◯	◯	◯	◯
Maternity/paternity leave impacted my salary/remuneration	◯	◯	◯	◯	◯	◯
Having a family changed my work hours/practice pattern	◯	◯	◯	◯	◯	◯

Please provide additional comments on how maternity/paternity leave has impacted advancement at work:

____

Lactation

Did you CHOOSE and/or PLAN to breastfeed any of your children?

◯ Yes
◯ No
◯ N/A

Which of your children did you breastfeed? (check all that apply)

☐ 1st child
☐ 2nd child
☐ 3rd child
☐ 4th child
☐ 5th child
☐ 6th child
(select all that apply)

Did you pump breastmilk at work as a resident?

◯ No
◯ Yes, for which child ______

Did you pump breastmilk at work as an attending staff?

◯ No
◯ Yes, for which child ______

How long did you pump at work (only the time while back at work, not in preparation for return)?

	< 1 month	1-3 months	3-6 months	>6months	N/A
1st child	◯	◯	◯	◯	◯
2nd child	◯	◯	◯	◯	◯
3rd child	◯	◯	◯	◯	◯
4th child	◯	◯	◯	◯	◯
5th child	◯	◯	◯	◯	◯
6th child	◯	◯	◯	◯	◯

What age was your first child when they stopped receiving direct breastfeeds (please enter in months of age)?

____
(in months)

What age was your second child when they stopped receiving direct breastfeeds (please enter in months of age)?

____
(in months)

What age was your third child when they stopped receiving direct breastfeeds (please enter in months of age)?

____
(in months)

What age was your fourth child when they stopped receiving direct breastfeeds (please enter in months of age)?

____
(in months)

What age was your fifth child when they stopped receiving direct breastfeeds (please enter in months of age)?

____
(in months)

What age was your sixth child when they stopped receiving direct breastfeeds (please enter in months of age)?

____
(in months)

What age was your first child when they stopped receiving pumped breastmilk (please enter age in months)?

____
(in months)

What age was your second child when they stopped receiving pumped breastmilk (please enter age in months)?

____
(in months)

What age was your third child when they stopped receiving pumped breastmilk (please enter age in months)?

____
(in months)

What age was your fourth child when they stopped receiving pumped breastmilk (please enter age in months)?

____
(in months)

What age was your fifth child when they stopped receiving pumped breastmilk (please enter age in months)?

____
(in months)

What age was your sixth child when they stopped receiving pumped breastmilk (please enter age in months)?

____
(in months)

Please state your agreement with the following statements:

	disagree	somewhat disagree	neutral	somewhat agree	agree
I had adequate time to pump at work	○	○	○	○	○
I had adequate space to pump at work (clean, private, accessible)	○	○	○	○	○
I had adequate space for pumped breastmilk storage at work	○	○	○	○	○
I met my breastfeeding goals	○	○	○	○	○
I felt supported in my decision to pump breastmilk at work	○	○	○	○	○
I experienced criticism or discrimination as a result of my need to pump breastmilk at work	○	○	○	○	○

Did you experience a breastfeeding complication (ie. reduced supply, blocked duct, mastitis) as a result of not being able to pump as frequently as you needed?

○ Yes
○ No

In your own words, please describe your experience pumping breastmilk at work:

____

Final Thoughts

Please list any topics that you would like to be addressed in future:

____

Additional comments:

____

L'identité de genre et son influence sur la planification familiale et l'avancement professionnel en otorhinolaryngologie - chirurgie tête et cou

Please click "Next Page" to begin the survey in French.

Données Démographiques

Quel est votre âge? (en années)

____

Quel est votre sexe?

- ◯ Agendre
- ◯ De genre queer ou au genre fluide
- ◯ Homme
- ◯ Non binaire
- ◯ En questionnement ou incertain(e)
- ◯ Bispirituel/bispirituelle
- ◯ Femme
- ◯ Préfère ne pas divulguer
- ◯ Autre catégorie de genre ______

Quelle est votre orientation sexuelle?

- ◯ Aromantique
- ◯ Asexué(e)
- ◯ Bisexuel(le)
- ◯ Fluide
- ◯ Gai
- ◯ Lesbienne
- ◯ Pansexuel(le)
- ◯ Queer
- ◯ En questionnement ou incertain
- ◯ Hommes noirs attirés par des personnes du même genre (APMG)
- ◯ Hétérosexuel(le)
- ◯ Préfère ne pas divulguer
- ◯ Autre catégorie non mentionnée (veuillez spécifier) ______

Quels pronoms utilisez-vous?

- ☐ Masculin
- ☐ Féminin
- ☐ Genre neutre
- ☐ Autre (veuillez spécifier) ______

(Veuillez choisir tous ceux qui s'appliquent à vous.)

Quelle est votre race/ethnicité?	☐ Asiatique de l'Est (Ex: Chinois(e), Japonais(e), Coréen(ne))<br>☐ Asiatique du Sud (ex: Indien(ne), Pakistanais(e), Sri-lankais(e))<br>☐ Asiatique du Sud-Est (ex: Malaisien(ne), Philippin(ne), Vietnamien(ne))<br>☐ Africain(ne) Noir(e) (ex: Ghanéen(ne), Kenyen(ne), Somalien(ne))<br>☐ Noir(e) des Caraïbes (ex: Barbadien(ne), Jamaïquain(ne))<br>☐ Noir(e) de l'Amérique du Nord (ex: Canadien(ne), Américain(ne))<br>☐ Premières Nations<br>☐ Caraïbe indien(ne) (ex: Guyanais(e) avec origine en Inde)<br>☐ Autochtone non mentionné ailleurs<br>☐ Inuit(e)<br>☐ Latino-Américain(ne) (ex: Argentin(e), Chilien(ne), Salvadorien(ne))<br>☐ Métis<br>☐ du Moyen-Orient (ex : Égyptien(ne), Iranien(ne), Libanais(e))<br>☐ Blanc de l'Europe (ex: Anglais(e), Italien(ne), Portugais(e), russe)<br>☐ Blanc de l'Amérique du Nord (Américain(ne), Canadien(ne))<br>☐ Patrimoine mixte (ex: Africain(ne) Noir(e) et Nord-Américain(e))<br>☐ Autre ______<br>☐ Préfère ne pas divulguer<br>☐ Incertain(e)<br>(Veuillez choisir tous ceux qui s'appliquent à vous.)
Quel est votre milieu de pratique?	◯ Académique<br>◯ Communautaire<br>◯ Communauté-académique
Quel est votre état civil?	◯ Marié(e)/Uni(e) civilement<br>◯ Célibataire<br>◯ En cohabitation<br>◯ Divorcé(e)/Séparé(e)<br>◯ Veuf/Veuve<br>◯ Préfère ne pas divulguer<br>◯ Autre, veuillez spécifier______
Dans quelle province habitez-vous?	◯ Alberta<br>◯ Colombie-Britannique<br>◯ Manitoba<br>◯ Nouveau-Brunswick<br>◯ Terre-Neuve<br>◯ Nouvelle-Écosse<br>◯ Ontario<br>◯ Île-du-Prince-Édouard<br>◯ Québec<br>◯ Saskatchewan<br>◯ Territoires du Nord-Ouest<br>◯ Nunavut<br>◯ Yukon

Quel est le stade de votre formation présentement?	◯ Résident/Fellow<br>◯ Consultant<br>◯ Retraité(e)
En quelle année de résidence êtes-vous?	◯ 1<br>◯ 2<br>◯ 3<br>◯ 4<br>◯ 5<br>◯ Fellow
Avez-vous l'intention de vous sous-spécialiser?	◯ Non/Otolaryngologie générale<br>◯ Chirurgie plastique et reconstruction faciale<br>◯ Chirurgie de la tête et du cou<br>◯ Laryngologie<br>◯ Otologie / Neurotologie<br>◯ Otolaryngologie pédiatrique<br>◯ Rhinologie<br>◯ Indécis(e)
Nombre d'années de pratique?	◯ Moins que 5<br>◯ 5-9<br>◯ 10-14<br>◯ 15-19<br>◯ 20 ou plus
En moyenne, combien de jours travaillez-vous par semaine (clinique ou autre)?	◯ 5 ou plus<br>◯ 3-4<br>◯ Moins que 3
Avez-vous eu recours à des technologies de reproduction assistée?	◯ Oui<br>◯ Non
Quelle est votre sous-spécialité principale?	◯ Générale<br>◯ Chirurgie plastique du visage<br>◯ Chirurgie de la tête et du cou<br>◯ Caryngologie<br>◯ Otologie<br>◯ Otolaryngologie pédiatrique<br>◯ Rhinologie
Quelle est votre sous-spécialité secondaire?	☐ Générale<br>☐ Chirurgie plastique du visage<br>☐ Chirurgie de la tête et du cou<br>☐ Laryngologie<br>☐ Otologie<br>☐ Otolaryngologie pédiatrique<br>☐ Rhinologie<br>☐ Aucun<br>(Veuillez choisir tous ceux qui s'appliquent à vous.)
Quel est votre rang académique actuel?	◯ Professeur(e) de l'enseignement supérieur<br>◯ Professeur adjoint(e)<br>◯ Professeur(e) agrégé(e)<br>◯ Professeur(e) titulaire<br>◯ Autre veuillez spécifier ______<br>◯ Aucun

Après combien d'années avez-vous atteint ce rang?
(inscrire le nombre d'années) __________________________________

Formation en Résidence

Quel était le pourcentage de collègues du même sexe que vous tout au cours de votre résidence?	◯ 0-20%<br>◯ 20-40%<br>◯ 40-60%<br>◯ 60-80%<br>◯ 80-100%
Quel était le pourcentage de personnel formateur du même sexe que vous durant votre résidence?	◯ 0-20%<br>◯ 20-40%<br>◯ 40-60%<br>◯ 60-80%<br>◯ 80-100%

Veuillez exprimer votre opinion face aux affirmations suivantes concernant les chefs de départements/services durant votre résidence:

	Fortement en désaccord	En désaccord	Neutre	Plutôt en accord	D'accord	N/A
Mes chefs de départements étaient favorables à l'idée que les résidents fondent une famille	○	○	○	○	○	○
Mon département était favorable à accorder un congé de maternité	○	○	○	○	○	○
Mon département était favorable à accorder un congé de paternité	○	○	○	○	○	○
Mon département était favorable à accorder des congés lors d'obligations familiales	○	○	○	○	○	○
Mon département avait les mêmes attentes des résidents sans égard à leur sexe	○	○	○	○	○	○
Les résidents étaient évalués équitablement, quel que soit leur sexe à partir des mêmes critères	○	○	○	○	○	○
Mes responsables de programmes traitaient tous les résidents de la même façon sans égard à leur sexe	○	○	○	○	○	○
On accordait les mêmes possibilités de leadership sans discrimination quant au genre	○	○	○	○	○	○

Veuillez exprimer votre opinion face aux affirmations suivantes concernant vos collègues de résidence durant votre formation:

	Fortement en désaccord	En désaccord	Neutre	Plutôt en accord	D'accord
Mes collègues de résidence de sexe féminin étaient favorables à l'idée que leurs consoeurs fondent une famille	◯	◯	◯	◯	◯
Mes collègues de résidence de sexe masculin étaient favorables à l'idée que leurs consoeurs fondent une famille	◯	◯	◯	◯	◯
Mes collègues de sexe féminin étaient favorables à la prise de congé pour des obligations familiales	◯	◯	◯	◯	◯
Mes collègues de sexe masculin étaient favorables à la prise de congé pour des obligations familiales	◯	◯	◯	◯	◯

Quelle cote accordez-vous à votre programme de formation dans son ensemble?

◯ Excellent
◯ Très bien
◯ Bien
◯ Passable
◯ Mauvaise

Selon vous, y aurait-il des améliorations à apporter à votre programme de résidence? Si oui, lesquelles?

____

Leadership

Quels postes de leadership avez-vous occupés (passé et présent)? Veuillez choisir tous ceux qui s'appliquent à vous.

☐ Chef de département
☐ Chef de site
☐ Chef de division
☐ Directeur de programme
☐ Directeur adjoint/directrice adjointe de programme
☐ Superviseur de rotation
☐ Autres ______
☐ Aucuns
(Veuillez choisir tous ceux qui s'appliquent à vous)

Quels pourcentages des rôles suivants sont occupés par des femmes dans votre département actuel?

	0-20%	20-40%	40-60%	60-80%	80-100%
Médecins	◯	◯	◯	◯	◯
Chef de département	◯	◯	◯	◯	◯

Chef de site	○	○	○	○	○
Chef de division	○	○	○	○	○
Directeur de programme	○	○	○	○	○
Directeur adjoint/Directrice adjointe de programme	○	○	○	○	○
Superviseur de rotations	○	○	○	○	○

Le Harcèlement Le harcèlement est une forme de discrimination. Il s'agit de tout comportement physique ou verbal indésirable qui choque ou humilie. En général, le harcèlement est un comportement qui persiste au fil du temps.

Les incidents ponctuels graves peuvent parfois aussi être considérés comme du harcèlement:

Il y a harcèlement lorsqu'une personne fait des remarques ou des blagues inopportunes sur votre race, votre religion, votre sexe, votre âge, votre handicap ou tout autre motif de discrimination vous menace ou vous intimide établit un contact physique inutile avec vous, comme vous toucher, vous tapoter, vous pincer ou vous frapper, ce qui peut également être considéré comme une agression Canadian Human Rights Commission (https://www.chrc-ccdp.gc.ca/eng/content/what-harassment-1)

Avez-vous subi du harcèlement lors du programme de formation/résidence?

○ Aucun harcèlement
○ Des nuances subtiles de harcèlement
○ Harcèlement perceptible
○ Harcèlement significatif
○ Incertain(e)

Types de harcèlement subi lors du programme de formation/résidence? (veuillez sélectionner toutes les réponses qui s'appliquent)

☐ Verbal (non sexuel)
☐ Harcèlement sexuel verbal
☐ Harcèlement sexuel physique
☐ Harcèlement racial/ethnique
☐ Harcèlement physique
(Veuillez choisir tous ceux qui s'appliquent à vous)

Qui était/étaient la/les personne(s) responsable(s) de harcèlement? (veuillez sélectionner toutes les réponses qui s'appliquent)

☐ Les chefs de département
☐ Des collègues ou autres résident(e)s
☐ Des patients ou leurs membres de famille
☐ Des membres du personnel auxiliaire
☐ Des membres de l'administration
(Veuillez choisir tous ceux qui s'appliquent à vous)

Avez-vous subi du harcèlement au travail?

○ Aucun harcèlement
○ Des nuances subtiles de harcèlement
○ Harcèlement perceptible
○ Harcèlement significatif
○ Incertain(e)

Veuillez sélectionner les types de harcèlement que vous avez subi après votre programme de formation/au travail

☐ Verbal (non sexuel)
☐ Harcèlement sexuel verbal
☐ Harcèlement sexuel physique
☐ Harcèlement racial/ethnique
☐ Harcèlement physique
(Veuillez choisir tous ceux qui s'appliquent à vous)

Qui était/étaient la/les personne(s) responsable(s) du harcèlement? (veuillez sélectionner toutes les réponses qui s'appliquent)	☐ Les chefs de département<br>☐ Des collègues ou autres résident(e)s<br>☐ Des patients ou leurs membres de famille<br>☐ Des membres du personnel auxiliaire<br>☐ Des membres de l'administration<br>(Veuillez choisir tous ceux qui s'appliquent à vous)
Veuillez élaborer sur votre expérience avec le harcèlement:	____

L'Équilibre Travail/Vie Personnelle Veuillez exprimer votre opinion face aux affirmations suivantes:

	Fortement en désaccord	En désaccord	Neutre	Plutôt en accord	D'accord
Je me sens à l'aise en tant que [gender_identity_f] au travail	◯	◯	◯	◯	◯
L'équilibre travail/vie personnelle est facile à maintenir	◯	◯	◯	◯	◯
Je suis satisfait(e) de ma décision de devenir otorhinolaryngologiste	◯	◯	◯	◯	◯
Je recommanderais l'otorhinolaryngologie comme carrière à un [gender_identity_f] débutant(e)	◯	◯	◯	◯	◯

Enfants, Congés de Maternité/Paternités, Lactation

Avez-vous des enfants?	◯ Oui<br>◯ Non<br>◯ Préfère ne pas répondre
Avez-vous déjà été enceinte?	◯ Oui<br>◯ Non<br>◯ Non applicable
Planifiez-vous avoir des enfants?	◯ Oui<br>◯ Non<br>◯ Incertain(e)
Avez-vous ou votre partenaire déjà eu une fausse couche?	◯ Oui combien? ______<br>◯ Non
Avez-vous ou votre partenaire donné naissance à un enfant, mort-né?	◯ Oui, combien? ______<br>◯ Non
Avez-vous ou votre partenaire subi un avortement thérapeutique?	◯ Oui, combien? ______<br>◯ Non
Avez-vous ou votre partenaire eu des signalements d'anomalies lors d'un dépistage prénatal?	◯ Oui<br>◯ Non
Veuillez élaborer au sujet de votre expérience avec des fausses-couches ou des complications lors d'une grossesse	________________________________

Veuillez exprimer votre opinion face aux affirmations suivantes :

	Fortement en désaccord	En désaccord	Neutre	Plutôt en accord	D'accord
Ma pratique a influencé ma décision d'avoir des enfants	◯	◯	◯	◯	◯
Ma pratique a influencé ma décision par rapport au moment à avoir des enfants	◯	◯	◯	◯	◯
Ma pratique a eu un impact sur ma capacité à avoir des enfants	◯	◯	◯	◯	◯
J'ai des préoccupations au sujet de ma planification familiale future	◯	◯	◯	◯	◯
J'ai des préoccupations au sujet de mon éventuelle fécondité	◯	◯	◯	◯	◯
J'ai des inquiétudes quant à l'avenir des congés de maternité/paternité	◯	◯	◯	◯	◯

Avez-vous déjà profité de services de fertilité?

◯ Oui
◯ Non

Avez-vous eu recours à des technologies de reproduction assistée?

◯ Oui
◯ Non

Si vous avez eu des difficultés avec la conception, quelle a été la raison médicale?

____

Veuillez élaborer au sujet de votre fécondité et planification familiale:

____

Combien d'enfants avez-vous?

____

Quel âge aviez-vous lorsque votre premier enfant est né?

____

À quel moment est-ce que vos enfants sont nés? (veuillez indiquer tous ceux qui s'appliquent à vous)

☐ Avant l'école de médecine
☐ Pendant l'école de médecine
☐ Pendant la formation/résidence/fellowship
☐ Pendant ma pratique
☐ Autre
(Veuillez choisir tous ceux qui s'appliquent à vous)

Avez-vous pris un congé de maternité/paternité lors de l'école de médecine, la formation/résidence ou la pratique?

◯ Oui
◯ Non

En moyenne, combien de semaines a duré votre congé de maternité/paternité lors de l'école de médecine?

____

Est-ce que votre congé de maternité/paternité a été aussi long que vous l'aviez prévu/planifié lors de l'école de médecine?

○ Oui
○ Non

En moyenne, combien de semaines a duré votre congé de maternité/paternité lors de votre formation/résidence?

____

Est-ce que votre congé de maternité/paternité a été aussi long que vous l'aviez prévu/planifié lors de votre formation/résidence?

○ Oui
○ Non

En moyenne, combien de semaines a duré votre congé de maternité/paternité lors de votre pratique?

____

Est-ce que votre congé de maternité/paternité a été aussi long que vous l'aviez prévu/planifié lors de votre pratique?

○ Oui
○ Non

Si vous avez un partenaire, est-ce que celui-ci a pris un congé de maternité/paternité?

○ Oui
○ Non
○ Non applicable

En moyenne, combien de semaines a duré le congé de paternité/maternité de votre partenaire?

____

Veuillez indiquer l'ampleur de l'influence des facteurs suivants sur la durée de votre congé:

	Aucun effet	Effet mineur	Neutre	Effet modéré	Effet profond	N/A
Support de votre partenaire/famille/amis	○	○	○	○	○	○
Préoccupations concernant la perte de compétences	○	○	○	○	○	○
Préoccupations concernant des opportunités futures	○	○	○	○	○	○
Préoccupations concernant la stigmatisation	○	○	○	○	○	○
La pression provenant de superviseurs	○	○	○	○	○	○
La pression provenant de collègues	○	○	○	○	○	○
Des préoccupations financières	○	○	○	○	○	○
De la difficulté à trouver un remplacement pour ma pratique	○	○	○	○	○	○
De la difficulté à trouver un service de garde pour mon/mes enfant(s)	○	○	○	○	○	○

S'ennuyer du travail/soigner des patients	◯	◯	◯	◯	◯	◯
Autre, veuillez préciser ______	◯	◯	◯	◯	◯	◯

La formation/la pratique a influencé le nombre d'enfants que j'ai CHOISI d'avoir.

- ◯ Fortement en désaccord
- ◯ En désaccord
- ◯ Neutre
- ◯ Plutôt en accord
- ◯ D'accord

La formation/la pratique a influencé ma capacité à avoir autant d'enfants que j'aurais voulus.

- ◯ Fortement en désaccord
- ◯ En désaccord
- ◯ Neutre
- ◯ Plutôt en accord
- ◯ D'accord

Avez-vous eu recours à des services de fertilité pour concevoir un enfant?

- ◯ Oui
- ◯ Non

Avez-vous eu recours à des technologies de reproduction assistée?

- ◯ Oui
- ◯ Non

Avez-vous choisi l'adoption?

- ◯ Oui
- ◯ Non

Si vous avez eu des difficultés avec la conception, quelle a été la raison médicale? ________________________________

Congé de Paternité/Maternité et Avancement

En moyenne, à combien de semaines de grossesse avez-vous arrêté les astreintes? ________________________________

Veuillez exprimer votre opinion face aux affirmations suivantes:

	Fortement en désaccord	En désaccord	Neutre	Plutôt en accord	D'accord	N/A
Mes collègues étaient favorables envers ma grossesse	◯	◯	◯	◯	◯	◯
Mes collègues étaient favorables envers mon congé de maternité/paternité	◯	◯	◯	◯	◯	◯
Le fait d'avoir eu un enfant a eu un impact sur ma décision de poursuivre mon fellowship ou de l'éducation post-programme additionnel	◯	◯	◯	◯	◯	◯

Le fait d'avoir eu un enfant a eu un impact sur ma décision de poursuivre des rôles de leadership au sein de mon département ou de mon lieu de travail	◯	◯	◯	◯	◯	◯
Mon congé de maternité/paternité a eu un impact sur le nombre d'opportunités d'avancement professionnel	◯	◯	◯	◯	◯	◯
Mon congé de maternité/paternité a eu un impact sur mon salaire	◯	◯	◯	◯	◯	◯
Le fait d'avoir une famille a changé mes heures de pratique/de travail	◯	◯	◯	◯	◯	◯

Veuillez élaborer sur comment votre congé de maternité/paternité a eu un impact sur votre avancement professionnel: ________________________________

Allaitement

Avez-vous choisi ou planifié d'allaiter vos enfants?
◯ Oui
◯ Non
◯ N/A

Lequel/lesquels de vos enfant(s) avez-vous allaité? (veuillez indiquer tous ceux qui s'appliquent)
☐ Premier enfant
☐ Deuxième enfant
☐ Troisième enfant
☐ Quatrième enfant
☐ Cinquième enfant
☐ Sixième enfant
(Veuillez choisir tous ceux qui s'appliquent à vous)

Avez-vous utilisé un tire-lait au travail pendant que vous étiez résident(e)?
◯ Non
◯ Oui, pour quel(s) enfant(s)? ______

Avez-vous utilisé un tire-lait au travail pendant que vous étiez docteur(e) traitant?
◯ Non
◯ Oui, pour quel(s) enfant(s)? ______

Combien de temps avez-vous tiré le lait maternel au travail même?

	< mois	1-3 mois	3-6 mois	> 6 mois	N/A
Premier enfant	◯	◯	◯	◯	◯
Deuxième enfant	◯	◯	◯	◯	◯
Troisième enfant	◯	◯	◯	◯	◯

Quatrième enfant	◯	◯	◯	◯	◯
Cinquième enfant	◯	◯	◯	◯	◯
Sixième enfant	◯	◯	◯	◯	◯

Quel âge avait votre premier enfant lorsqu'il a reçu son dernier allaitement (veuillez inscrire l'âge de votre enfant en mois)? ____________ (en mois)

Quel âge avait votre deuxième enfant lorsqu'il a reçu son dernier allaitement (veuillez inscrire l'âge de votre enfant en mois) ? ____________ (en mois)

Quel âge avait votre troisième enfant lorsqu'il a reçu son dernier allaitement (veuillez inscrire l'âge de votre enfant en mois)? ____________ (en mois)

Quel âge avait votre quatrième enfant lorsqu'il a reçu son dernier allaitement (veuillez inscrire l'âge de votre enfant en mois)? ____________ (en mois)

Quel âge avait votre cinquième enfant lorsqu'il a reçu son dernier allaitement (veuillez inscrire l'âge de votre enfant en mois)? ____________ (en mois)

Quel âge avait votre sixième enfant lorsqu'il a reçu son dernier allaitement (veuillez inscrire l'âge de votre enfant en mois)? ____________ (en mois)

Quel âge avait votre premier enfant lorsqu'il a reçu son dernier allaitement de lait maternel pompé? (veuillez inscrire l'âge de votre enfant en mois) ____________ (en mois)

Quel âge avait votre deuxième enfant lorsqu'il a reçu son dernier allaitement de lait maternel pompé? (veuillez inscrire l'âge de votre enfant en mois) ____________ (en mois)

Quel âge avait votre troisième enfant lorsqu'il a reçu son dernier allaitement de lait maternel pompé? (veuillez inscrire l'âge de votre enfant en mois) ____________ (en mois)

Quel âge avait votre quatrième enfant lorsqu'il a reçu son dernier allaitement de lait maternel pompé? (veuillez inscrire l'âge de votre enfant en mois) ____________ (en mois)

Quel âge avait votre cinquième enfant lorsqu'il a reçu son dernier allaitement de lait maternel pompé? (veuillez inscrire l'âge de votre enfant en mois) ____________ (en mois)

Quel âge avait votre sixième enfant lorsqu'il a reçu son dernier allaitement de lait maternel pompé? (veuillez inscrire l'âge de votre enfant en mois) ____________ (en mois)

Veuillez exprimer votre opinion face aux affirmations suivantes:

	Fortement en désaccord	En désaccord	Neutre	Plutôt en accord	D'accord
J'avais le temps adéquat pour la collecte de lait maternel sur le lieu de travail	○	○	○	○	○
J'avais l'espace adéquat pour la collecte de lait maternel sur le lieu de travail	○	○	○	○	○
J'avais l'espace adéquat pour stocker mon lait maternel au travail	○	○	○	○	○
J'ai atteint mes objectifs personnels d'allaitement	○	○	○	○	○
Je me suis senti(e) soutenu(e) dans ma décision de tirer le lait maternel au travail	○	○	○	○	○
J'ai subi des critiques ou de la discrimination au travail à cause du besoin de tirer le lait maternel	○	○	○	○	○

Avez-vous éprouvé un de ces problèmes d'allaitement (blocage de canaux lactifères, une mastite ou une quantité de lait réduite) à cause de l'impossibilité de tirer le lait maternel assez souvent?

○ Oui
○ Non

Dans vos propres mots, veuillez décrire votre expérience avec le tirage de lait maternel au travail:

____

Dernières Pensées

Veuillez noter d'autres sujets que vous aimeriez aborder à l'avenir:

____

Commentaires additionnels:

____
